# Using individual preferences and BWM with entropy measures to modify the Global Entrepreneurship Index

**DOI:** 10.1371/journal.pone.0266187

**Published:** 2022-03-25

**Authors:** Caixiang Chen, An Zhu

**Affiliations:** 1 Logistics and e-Commerce College, Zhejiang Wanli University, Ningbo, PR China; 2 Faculty of Design and Architecture, Zheliang Wanli University, Ningbo, PR China; LUMSA: Libera Universita Maria Santissima Assunta, ITALY

## Abstract

The Global Entrepreneurship and Development Institute (GEDI) annually releases the Global Entrepreneurship Index (GEI) to measure the quality and dynamics of entrepreneurship ecosystems at a national and regional level. The published Global Entrepreneurship Index takes the arithmetic mean value of the individual level of entrepreneurial attitudes, abilities and aspirations. In this paper, we alternatively consider all individual preferences among these three sub-indices, the performance results of which are obtained by means of a sophisticated manner. The entropy values of these performance results are derived to reduce the information redundancy. The best-worst method (BWM) is employed to determine the common weights with respect to each individual preference. An empirical study using the data of GEI-2019 is performed to indicate the validness of our methodology.

## 1. Introduction

The economic value of entrepreneurship has been intensively investigated in literature. Van Praag and Versloot presented a systematic review about the empirical studies that answers the following question: what is the contribution of entrepreneurs to the economy compared to non-entrepreneurs [1]. Van Stel et al. concluded that the entrepreneurship plays a different role in countries at the different stages of economic development [2]. Valliere and Peterson suggested that, in developed countries, the entrepreneurship has a significant effect on the economic growth. However, such an effect is absent in the emerging countries [3]. Hessels and van Stel further indicated that the export-oriented early-stage entrepreneurship has an additional positive effect on macroeconomic growth in higher-income countries. However, there is no such an additional effect in lower-income countries [4]. Du and O’Connor claimed that the new product entrepreneurship and to a lesser extent improvement-driven opportunity entrepreneurship both significantly contribute to the improvement of national level efficiency [5]. Sutter et al. argued that entrepreneurship is a critical means of extreme poverty alleviation. Although the significance of entrepreneurship has been recognized in both academic research and practice, it is less surprising that the measurement of entrepreneurship is far from easy. The need for comprehensive measures of entrepreneurial performance is evident across governments and in the field of public policy monitoring and evaluation [6]. Decision makers increasingly rely on charts and rankings that compare national and regional entrepreneurship ecosystems with one another. The Global Entrepreneurship and Development Institute (GEDI) annually reports the Global Entrepreneurship Index (GEI) to measure the quality and dynamics of entrepreneurship ecosystems at a national and regional level since 2009. The published GEI combines the individual level of entrepreneurial attitudes, abilities and aspirations into a composite indicator, and provides policymakers with a tool to better understand the entrepreneurial strengths and weaknesses of their countries and regions. Entrepreneurial attitudes are societies’ attitudes towards entrepreneurship, which are defined by GEDI as “a population’s general feelings about recognizing opportunities, knowing entrepreneurs personally, endowing entrepreneurs with high status, accepting the risks associated with business startups, and having the skills to launch a business successfully”. Entrepreneurial abilities are defined as “startups in the medium- or high-technology sectors that are initiated by educated entrepreneurs, and launched because of someone being motivated by an opportunity in an environment that is not overly competitive”. Entrepreneurial aspirations are defined as “the early-stage entrepreneur’s effort to introduce new products and/or services, develop new production processes, penetrate foreign markets, substantially increase their company’s staff, and finance their business with formal and/or informal venture capital”. Each of these three sub-indices influences the other two. For instance, entrepreneurial attitudes significantly influence entrepreneurial abilities and entrepreneurial aspirations, meanwhile, entrepreneurial abilities and entrepreneurial aspirations also influence entrepreneurial attitudes. The aforementioned three sub-indices contain 14 pillars of entrepreneurship, each of which includes an individual and an institutional variable that is tightly linked to the micro- and the macro-level aspects of entrepreneurship, and is summarized by GEDI as below:

- Entrepreneurial Attitudes Pillars: Opportunity Perception, Startup Skills, Risk Acceptance, Networking and Cultural Support;
- Entrepreneurial Abilities Pillars: Opportunity Startup, Technology Absorption, Human Capital and Competition;
- Entrepreneurial Aspirations Pillars: Product Innovation, Process Innovation, High Growth and Internationalization.

More specifically, the details of aforementioned 14 pillars are given as below:

- Opportunity Perception: This pillar captures the potential “opportunity perception” of a population by considering the state of property rights and the regulatory burden that could limit the real exploitation of the recognized entrepreneurial opportunity.
- Startup Skills: Skill Perception measures the percentage of the population who believe they have adequate startup skills.
- Risk Acceptance: Risk Perception is defined as the percentage of the population who do not believe that fear of failure would prevent them from starting a business.
- Networking: Networking combines an entrepreneur’s personal knowledge with their ability to connect to others in a country and the whole world.
- Cultural Support: This pillar is a combined measure of how a country’s inhabitants view entrepreneurs in terms of status and career choice, and how the level of corruption in that country affects this view.
- Opportunity Startup: This is a measure of startups by people who are motivated by opportunity but face red tape and tax payment.
- Technology Absorption: This is a measure of a country’s capacity for firm-level technology absorption, as reported by the World Economic Forum.
- Human Capital: Labor Freedom measures the freedom of the labor from the regulatory perspective and Staff Training is a country’s level of investment in business training and employee development.
- Competition: Competition is a measure of a business’s product or market uniqueness, combined with the market power of existing businesses and business groups and the effectiveness of anti-monopoly regulation.
- Product Innovation: Technology Transfer is a complex measure of whether a business environment allows the application of innovations for developing new products.
- Process Innovation: An appropriate institutional variable applied here is complex measure combining research and development (R&D), the quality of scientific institutions in a country (Scientific Institutions) and the availability of scientists and engineers (Availability of Scientist).
- High Growth: High Growth is a combined measure of the percentage of high-growth businesses that intend to employ at least 10 people and plan to grow more than 50 percent in five years (Gazelle variable) with business strategy sophistication (Business Strategy variable) and venture capital financing possibility (Venture Capital).
- Internationalization: The internationalization pillar is designed to capture the degree to which a country’s entrepreneurs are internationalized, as measured by the exporting potential of businesses, controlling for the extent to which the country is able to produce complex products.
- Risk Capital: The informal investment (Informal Investment) and the institutional depth of capital market (DCM).

All these pillars are an attempt to capture the open-ended nature of entrepreneurship. Zoltán J Ács et al. claimed that entrepreneurship should be treated as a systematic phenomenon, and adopting a systemic approach to measuring the country-wide entrepreneurial performance is significant and meaningful [7]. By doing so, not only an in-depth view of the strengths and weaknesses of the entrepreneurship could be outlined, but also academic researchers and policy- makers is capable of taking a broad standpoint when examining individual- and country-level indicators of entrepreneurial action.

The GEI has been widely recognized as an effective tool to provide a solid-theory-based entrepreneurship measure. Bonyadi and Sarreshtehdari conducted a critical review of the computation methods of the GEI, with the aid of assessing the shortfalls in the model used for the calculation of the GEI [8]. Zoltán J. Ács et al. introduced a novel concept of National Systems of Entrepreneurship (NSEs) and provided a novel index methodology to characterize NSEs [7]. Zoltán J. Ács et al. proposed a regional application of the Global Entrepreneurship and Development Index (GEDI) that captures the contextual features of entrepreneurship across regions [9]. However, based upon the efficiency analysis of the GEI, Junior et al. indicated that the Key Performance Indicators’ analysis results in a misinterpretation of the dynamics of NSEs [10].

Another popular method to measure entrepreneurship across national and regional contexts is Total Entrepreneurial Activity (AET) index based upon the annual surveys reported by the Global Entrepreneurship Monitor (GEM) Consortium. The measurement and comparative analysis of national entrepreneurship is a relatively new and under-represented area of academic research. Baker et al. developed a Comparative Discovery, Evaluation and Exploitation (CDEE) framework for comparing entrepreneurship processes across nations [11]. Marcotte reviewed and analyzed the existing entrepreneurship indices from the standpoints of conceptual and methodological dimensions [12]. Terjesen et al. systematically reviewed and assessed the current status of research Comparative International Entrepreneurship and highlighted the research gaps and suggested promising research directions [13].

Although this small body of research is helpful to effectively guide comparative analysis and directions of innovation and enforcement strategies, it is crucial to develop a sophisticated mechanism to improve the published GEI, since the present GEI takes the arithmetic mean of the three sub-indices, and inevitably neglects the fact that different decision makers may have different preferences among these sub-indices [14, 15]. Relative the weakness associated with the arithmetic mean calculation, Szerb László [16] and Zoltán J Ács [17] developed the Penalty for Bottleneck (PfB) methodology to re-build the GEI. However, the PfB-adjusted GEI could not be an optimal solution because of the unknown magnitude of that penalty [9]. In the case of decision making by management committees and social choice problems, it is not uncommon that the preferences among multiple sub-indices exhibit a substantial degree of variability [18]. When it is impossible to ignore any individual preference completely, the best choice is to accept all possible preferences first, and then combine the results obtained from different preferences [19]. However, the extant literature developing entrepreneurship indices has left this topic largely unexplored. This study fills the void by considering all possible preferences among these sub-indices released by the GEDI, and then develops a best-worst method (BWM) with entropy measures to determine a set of common weights associated with each preference.

In contrast with previous papers that using either arithmetic or geometric mean to aggregate the entrepreneurship performance, this study provides a new framework to measure and compare the entrepreneurship across national and regional contexts. The main contributions of this work are twofold. First, we characterize and quantify the individual preferences among the sub-indices of the GEI in a sophisticated manner, which thereby could be utilized to formulate a new decision matrix for modifying the GEI. Second, we employ the Shannon entropy in information theory to reduce the information redundancy of the decision matrix. Third, the BWM is applied to elicit a set of common weights associated with each preference for modifying the GEI, along with the sensitivity analysis to provide academic, managerial and policy-related implications.

The rest of this paper proceeds as follows. The methodology formulation is proposed in Section 2. A case study using the released data in 2019 is provided in Section 3. Concluding remarks are offered in Section 4.

## 2. Methodology formulation

The GEI not only presents a detailed insight on the health condition of entrepreneurial ecosystems across countries and regions, but also provides leaders with the process of developing new polices that accelerate new firm formation, innovation and job creation. According to the definition, the GEI takes into account various measures of entrepreneurial attitudes (ATT), entrepreneurial abilities (ABT), and entrepreneurial aspirations (ASP). That is,

$$GEI=\frac{ATT+ABT+ASP}{3}$$

The GEDI also annually reports the rankings in terms of the GEI and the sub-indices.

The methodology proposed is two-fold and begins with investigating the individual preferences, along with the construction of a new decision matrix; we then compute the entropy measures of that decision matrix and lastly apply the BWM to determine the common weights associated with each individual preference.

### 2.1. Individual preference

In order to modify the GEI, we completely consider all possible preferences among ATT, ABT and ASP, that is, *ATT*≻*ABT*≻*ASP*(*TBS*), *ATT*≻*ASP*≻*ABT*(*TSB*), *ABT*≻*ATT*≻*ASP*(*BTS*), *ABT*≻*ASP*≻*ATT*(*BST*), *ASP*≻*ATT*≻*ABT*(*STB*), *ASP*≻*ABT*≻*ATT*(*SBT*). Each of these preferences is denoted by a mild weight restriction, for instance, the preference TBS is represented by *w*_*i*1_≥*w*_*i*2_≥*w*_*i*3_, *w*_*ij*_≥0. For the comparable purpose, we normalize the values of ATT (*x*_*i*1_), ABT (*x*_*i*2_) and ASP (*x*_*i*3_) into 0–1 scale using:

$$y_{ij}=\frac{x_{ij}}{\sum_{i=1}^{m}x_{ij}},\;i=1,2,\ldots ,m,\;j=1,2,3$$ (1)

The performance of country i is denoted as a weighted sum of the performance measures under each sub-indices. With loss of generality, we first investigate the preference TBS as:

$$\begin{array}{l} S_{i}=\max\sum_{j=1}^{3}y_{ij}w_{ij}\\ s.t.\;w_{i1}\geq w_{2i}\geq w_{i3}\\ \;\sum_{j=1}^{3}w_{j}=1,w_{j}\geq 0 \end{array}$$ (2)

We denote *v*_*i*1_ = *w*_*i*1_−*w*_*i*2_≥0, *v*_*i*2_ = *w*_*i*2_−*w*_*i*3_≥0, *v*_*i*3_ = *w*_*i*3_≥0, and obtain

$$\begin{array}{ccc} \sum_{j=1}^{3}w_{ij} & = & (w_{i1}-w_{i2})+2(w_{i2}-w_{i3})+3w_{i3}\\ & = & \sum_{j=1}^{3}jv_{ij}\\ & = & 1 \end{array}$$

We also introduce $\phi_{ij}=\sum_{t=1}^{j}y_{it}$, and then have

$$\begin{array}{ccc} \sum_{j=1}^{3}y_{ij}w_{ij} & = & \left(w_{i1}-w_{i2})y_{i1}+(w_{i2}-w_{i3})(y_{i1}+y_{i2})+w_{i3}(y_{i1}+y_{i2}+y_{i3}\right)\\ & = & \phi_{i1}v_{i1}+\phi_{i2}v_{i2}+\phi_{i3}v_{i3}\\ & = & \sum_{j=1}^{3}\phi_{ij}v_{ij} \end{array}$$

Therefore, the mathematical model (2) is equivalent to the following formulation:

$$\begin{array}{l} S_{i}=\max\sum_{j=1}^{3}v_{ij}\phi_{ij}\\ s.t.\;\sum_{j=1}^{3}jv_{ij}=1,\\ \;v_{i1}\geq 0,v_{i2}\geq 0,v_{i3}\geq 0 \end{array}$$ (3)

The dual of (3) is

$$\begin{array}{l} \min\;z_{i}\\ s.t.\;z_{i}\geq \frac{1}{j}\phi_{ij} \end{array}$$ (4)

The optimal objective value of (4) is reasonably obtained at the points $z_{i}=\max\{\varphi_{i1},\frac{\varphi_{i2}}{2},\frac{\varphi_{i3}}{3}\}=\max\{y_{i1},\frac{y_{i1}+y_{i2}}{2},\frac{y_{i1}+y_{i2}+y_{i3}}{3}\}$, which is also the optimal value of (2).

The performance of countries under other preferences can be easily obtained in the same manner. We summarize the results under all preferences in Table 1 below and the decision matrix *S*_*m*6_.

**Table 1:** Results under all preferences.

Preferences	Results
TBS	$\max\{y_{i1},\frac{y_{i1}+y_{i2}}{2},\frac{y_{i1}+y_{i2}+y_{i3}}{3}\}$
TSB	$\max\{y_{i1},\frac{y_{i1}+y_{i3}}{2},\frac{y_{i1}+y_{i2}+y_{i3}}{3}\}$
BTS	$\max\{y_{i2},\frac{y_{i1}+y_{i2}}{2},\frac{y_{i1}+y_{i2}+y_{i3}}{3}\}$
BST	$\max\{y_{i2},\frac{y_{i1}+y_{i3}}{2},\frac{y_{i1}+y_{i2}+y_{i3}}{3}\}$
STB	$\max\{y_{i3},\frac{y_{i1}+y_{i2}}{2},\frac{y_{i1}+y_{i2}+y_{i3}}{3}\}$
SBT	$\max\{y_{i3},\frac{y_{i1}+y_{i3}}{2},\frac{y_{i1}+y_{i2}+y_{i3}}{3}\}$


$$S_{m6}=[\begin{array}{cccccc} S_{1}^{1} & S_{1}^{2} & S_{1}^{3} & S_{1}^{4} & S_{1}^{5} & S_{1}^{6}\\ S_{2}^{1} & S_{2}^{2} & S_{2}^{3} & S_{2}^{4} & S_{2}^{5} & S_{2}^{6}\\ \vdots & \vdots & \vdots & \vdots & \vdots & \vdots \\ S_{m}^{1} & S_{m}^{2} & S_{m}^{3} & S_{m}^{4} & S_{m}^{5} & S_{m}^{6} \end{array}]$$ (5)

The superscripts {1,2,3,4,5,6} in (5) represent the aforementioned preferences TBS, TSB, BTS, BST, STB and SBT, respectively. $S_{i}^{k}$, *i* = 1,2,…,*m*, *k* = 1,2,…,6, exhibits the performance of country *i* under preference *k*.

### 2.2. BWM with entropy measures

As an effective mathematical concept to measure uncertainty, the Shannon entropy is employed to reduce the information redundancy of the decision matrix [20]. After normalizing the decision matrix *S*_*m*6_, then entropy values of the elements of Sm6 is defined as

$$e_{ik}=-s_{i}^{k}\ln s_{i}^{k}$$ (6)

where $s_{i}^{k}=\frac{S_{i}^{k}}{\sum_{i=1}^{m}S_{i}^{k}}$. Then the above decision matrix *S*_*m*6_ turns into ⌊*e*_*ij*_⌋_*m*6_, which is proposed to be solved by the Best-worst method (BWM).

The BWM is a multi-criteria decision-making (MCDM) method that utilizes two vectors of pairwise comparisons to elicit the weights of criteria [21, 22]. The best (most important) and worst (least important) criteria are identified firstly by the decision maker, after which pairwise comparisons are performed between each of these two criteria (best and worst) and the other criteria. A non-linear model [21] and a linear model [22] are respectively formulated and solved to determine the weights with respect to various criteria.

In the context of modifying the GEI, a pairwise comparison of 6 individual preferences is conducted using a 1/9 to 9 scale. In line with the Analytic Hierarchy Process (AHP) introduced by Saaty [23], the relative importance between two individual preferences is measured using a numerical scale from 1 to 9. It is also possible to assign intermediate values that do not correspond to a precise interpretation. The pairwise comparison matrix could be derived as

$$A={\left(a_{ih}\right)}_{6\times 6}=(\begin{array}{cccc} a_{11} & a_{12} & \cdots & a_{16}\\ a_{21} & a_{22} & \cdots & a_{26}\\ \vdots & \vdots & \ddots & \vdots \\ a_{61} & a_{62} & \cdots & a_{66} \end{array})$$ (7)

in which *a*_*tk*_ indicates the relative preference of individual preference *t* to individual preference *hk*, $a_{kt}=\frac{1}{a_{tk}}$, and *a*_*tt*_ = 1. Specifically, *a*_*tk*_ = 1 demonstrates that *t* and *k* are of the same importance. *a*_*tk*_>1 represents that *t* is more important than *k*. The pairwise comparison matrix is perfectly consistent when *a*_*tl*_×*a*_*lk*_ = *a*_*tk*_, ∀*t*, *k*.

1. Determine the best and worst individual preferences.
2. Determine the preference of the best individual preference over all the other individual preferences using a scale between 1 and 9. The resulting Best-to-Others vector is *A*_*B*_ = (*a*_*B*1_,*a*_*B*2_,…,*a*_*B*6_), in which *a*_*Bk*_, *k* = 1,2,…,6 shows the preference of the best individual preference over individual preference k.
3. Determine the preference of all the individual preferences over all the worst individual preference using a scale between 1 and 9. The resulting Others-to-Worst vector is *A*_*W*_ = (*a*_1*W*_,*a*_2*W*_,…,*a*_6*W*_)^*T*^. in which *a*_*kW*_, *k* = 1,2,…,6 shows the preference of individual preference *k* over the worst individual preference.
4. Find the optimal weights. The optimal weights associated with different individual preferences should be determined to minimize the maximum absolute differences $\left|\frac{\lambda_{B}}{\lambda_{k}}-a_{Bk}\right|$ and $\left|\frac{\lambda_{k}}{\lambda_{W}}-a_{kW}\right|$, for all *k*. This could be translated to a minmax model below:

$$\begin{array}{l} \min\;\max_{k}\left\{|\frac{\lambda_{B}}{\lambda_{k}}-a_{Bj}|,|\frac{\lambda_{k}}{\lambda_{W}}-a_{kW}|\right\}\\ s.t.\;\sum_{k=1}^{6}\lambda_{k}=1,\lambda_{k}\geq 0 \end{array}$$ (8)

This non-linear program is equivalent to the following expression with 2×6 constraints:

$$\begin{array}{cc} & \min\;\xi \\ s.t. & |\frac{\lambda_{B}}{\lambda_{k}}-a_{Bj}|\leq \xi \\ & |\frac{\lambda_{k}}{\lambda_{W}}-a_{kW}|\leq \xi \\ & \sum_{k=1}^{6}\lambda_{k}=1,\lambda_{k}\geq 0 \end{array}$$ (9)

Since the non-linear model may give rise to multiple optimal solutions, Rezaei transfers it into a linear program for providing a unique solution [22]:

$$\begin{array}{cc} & \min\;\zeta \\ s.t. & |\lambda_{B}-a_{Bk}\lambda_{k}|\leq \zeta \\ & |\lambda_{k}-a_{kW}\lambda_{W}|\leq \zeta \\ & \sum_{k=1}^{6}\lambda_{k}=1,\lambda_{k}\geq 0 \end{array}$$ (10)

The optimal weights with respect to different importance orders $\left(\lambda_{1}^{*},\lambda_{2}^{*},\ldots ,\lambda_{6}^{*}\right)$ and ζ* can be obtained by solving the linear problem (10) with 2×6 constraints. In addition, ζ* can be regarded as an index of consistence of the comparisons. The following Table 2 reports the maximum value of consistence index *ζ* for different values of *a*_*BW*_.

**Table 2:** Consistence index table (Rezaei, 2015).

*a*_*BW*_	1	2	3	4	5	6	7	8	9
Consistence index	0	0.44	1	1.63	2.30	3	3.73	4.47	5.23

In this manner, Rezaei (2015, 2016) propose a consistency ratio as

$$\mathrm{consistenc}e\;\mathrm{ratio}=\frac{\zeta^{*}}{\mathrm{consistence}\;\mathrm{index}}$$

Clearly, the values of consistence ratio approximate to 0 indicate more consistency, while the values approximate to 1 mean less consistency. Therefore, the smaller the ζ* is, the more consistent the comparisons will be. A salient characteristic of the BWM is the generation of more reliable results, in terms of more consistent comparisons.

Consequently, the ultimate evaluation results associated with each country or region derived from the proposed methodology is:

$$S_{i}^{*}=\sum_{k=1}^{6}\lambda_{k}^{*}e_{i}^{k},\;i=1,2,\ldots ,m$$ (11)

## 3. Case study

### 3.1. Data source

To investigate the validness and effectiveness of the proposed methodology, we conduct an empirical study to modify the Global Entrepreneurship Index using the data of GEI-2019, which reports the rankings of 137 countries/regions, and provides confidence intervals for the GEI. The G20 (or Group of Twenty) is an intergovernmental forum comprising 19 countries and the European Union (EU). The EU is represented by the European Commission and the European Central Bank. Collectively, the G20 economies account for around 90% of the gross world product (GWP), 80% of international trade (or, if excluding EU intra-trade, 75%), two-thirds of the world population, and approximately half of the world’s land area. 19 individual countries of G20 are chosen to illustrate our methodology. The ATT, ABT, ASP and GEI values of these 19 individual countries of G20 are reported in the Table 3 below, in which the countries are alphabetically listed.

**Table 3:** The GEI-2019 data for 19 G20 countries.

Country	ATT	ABT	ASP	GEI	Normalized ATT	Normalized ABT	Normalized ASP
Argentina	27.9	24.4	25.7	26.0	0.0312	0.0253	0.0293
Australia	80.1	65.2	74.1	73.1	0.0896	0.0675	0.0844
Brazil	15.6	8.3	24.5	16.1	0.0174	0.0086	0.0279
Canada	83.8	79.4	78.0	80.4	0.0937	0.0823	0.0888
China	34.2	66.6	36.8	45.9	0.0383	0.0690	0.0419
France	66.8	77.7	56.8	67.1	0.0747	0.0805	0.0647
Germany	68.2	74.0	57.8	66.7	0.0763	0.0767	0.0658
India	23.6	28.9	22.7	25.1	0.0264	0.0299	0.0259
Indonesia	28.4	17.2	32.3	26.0	0.0318	0.0178	0.0368
Italy	40.5	57.0	37.9	45.1	0.0453	0.0590	0.0432
Japan	61.4	67.1	31.4	53.3	0.0687	0.0695	0.0358
Mexico	25.0	25.9	30.4	27.1	0.0280	0.0268	0.0346
Russia	27.6	19.6	27.0	24.7	0.0309	0.0203	0.0308
Saudi Arabia	29.8	39.6	56.8	42.1	0.0333	0.0410	0.0647
South Africa	29.3	39.2	26.3	31.6	0.0328	0.0406	0.0300
South Korea	46.3	60.1	67.8	58.1	0.0518	0.0623	0.0772
Turkey	33.2	51.6	34.6	39.8	0.0371	0.0535	0.0394
United Kingdom	82.6	76.3	73.5	77.5	0.0924	0.0790	0.0837
United States	89.7	87.2	83.5	86.8	0.1003	0.0903	0.0951

### 3.2. Results and discussion

In light of the fact that the GEI is composed of ATT, ABT and ASP, we reasonably identify six (A3 = 6) individual preferences among them. That are, *TBS*:*ATT*≻*ABT*≻*ASP*, *TSB*:*ATT*≻*ASP*≻*ABT*, *BTS*:*ABT*≻*ATT*≻*ASP*, *BST*:*ABT*≻*ASP*≻*ATT*, *STB*:*ASP*≻*ATT*≻*ABT*, *SBT*: *ASP*≻*ABT*≻*ATT*. For each individual preference, the optimal decision results associated with each country could be derived in a closed-form, without the determination of the precise weights of ATT, ABT and ASP, which are presented in the following Table 4.

**Table 4:** The new decision matrix.

Country	TBS	TSB	BTS	BST	STB	SBT
Argentina	0.0312	0.0312	0.0286	0.0286	0.0302	0.0293
Australia	0.0896	0.0896	0.0805	0.0805	0.0870	0.0844
Brazil	0.0180	0.0227	0.0180	0.0183	0.0279	0.0279
Canada	0.0937	0.0937	0.0883	0.0883	0.0913	0.0888
China	0.0536	0.0497	0.0690	0.0690	0.0497	0.0555
France	0.0776	0.0747	0.0805	0.0805	0.0733	0.0733
Germany	0.0765	0.0763	0.0767	0.0767	0.0729	0.0729
India	0.0282	0.0274	0.0299	0.0299	0.0274	0.0279
Indonesia	0.0318	0.0343	0.0288	0.0288	0.0368	0.0368
Italy	0.0522	0.0492	0.0590	0.0590	0.0492	0.0511
Japan	0.0691	0.0687	0.0695	0.0695	0.0580	0.0580
Mexico	0.0298	0.0313	0.0298	0.0307	0.0346	0.0346
Russia	0.0309	0.0309	0.0273	0.0273	0.0308	0.0308
Saudi Arabia	0.0464	0.0490	0.0464	0.0529	0.0647	0.0647
South Africa	0.0367	0.0344	0.0406	0.0406	0.0344	0.0353
South Korea	0.0638	0.0645	0.0638	0.0697	0.0772	0.0772
Turkey	0.0453	0.0433	0.0535	0.0535	0.0433	0.0464
United Kingdom	0.0924	0.0924	0.0857	0.0851	0.0881	0.0851
United States	0.1003	0.1003	0.0953	0.0953	0.0977	0.0953

According to (6), we calculate the entropy values of Table 4 and obtain a revised decision matrix below (See Table 5).

**Table 5:** The revised decision matrix.

Country	TBS	TSB	BTS	BST	STB	SBT
Argentina	0.1033	0.1035	0.0967	0.0959	0.1005	0.0981
Australia	0.2080	0.2084	0.1945	0.1931	0.2035	0.1998
Brazil	0.0688	0.0820	0.0686	0.0688	0.0948	0.0948
Canada	0.2137	0.2141	0.2057	0.2042	0.2095	0.2060
China	0.1503	0.1432	0.1766	0.1753	0.1422	0.1529
France	0.1906	0.1866	0.1945	0.1931	0.1832	0.1831
Germany	0.1889	0.1890	0.1887	0.1873	0.1826	0.1825
India	0.0960	0.0942	0.1000	0.0991	0.0935	0.0947
Indonesia	0.1046	0.1107	0.0972	0.0964	0.1155	0.1155
Italy	0.1476	0.1421	0.1598	0.1585	0.1411	0.1448
Japan	0.1773	0.1769	0.1775	0.1761	0.1575	0.1575
Mexico	0.1000	0.1037	0.0997	0.1010	0.1107	0.1106
Russia	0.1025	0.1027	0.0936	0.0927	0.1018	0.1017
Saudi Arabia	0.1363	0.1418	0.1359	0.1473	0.1692	0.1691
South Africa	0.1159	0.1111	0.1241	0.1230	0.1103	0.1121
South Korea	0.1684	0.1700	0.1679	0.1765	0.1892	0.1892
Turkey	0.1341	0.1304	0.1496	0.1484	0.1295	0.1357
United Kingdom	0.2119	0.2122	0.2021	0.1997	0.2050	0.2007
United States	0.2223	0.2227	0.2153	0.2137	0.2180	0.2147

In the presence of the above revised decision matrix, we provide the pairwise comparison vectors as shown in Table 6. Solving this problem by means of the linear program (10) generates $\lambda_{TBS}^{*}=0.0645$, $\lambda_{TSB}^{*}=0.0968$, $\lambda_{BTS}^{*}=0.1935$, $\lambda_{BST}^{*}=0.1290$, $\lambda_{STB}^{*}=0.1613$, $\lambda_{STB}^{*}=0.3548$, and ζ* = 0.0323. ζ* = 0.0323 indicates that this comparison system is sufficiently consistent.

**Table 6:** Best-to-Others (BO) and Others-to-Worst (OW) pairwise comparison vectors.

BO: Best SBT	TBS	TSB	BTS	BST	STB	SBT
OW						Worst: TBS
TBS						
TSB						
BTS						
BST						
STB						
SBT						

**Fig 1:**
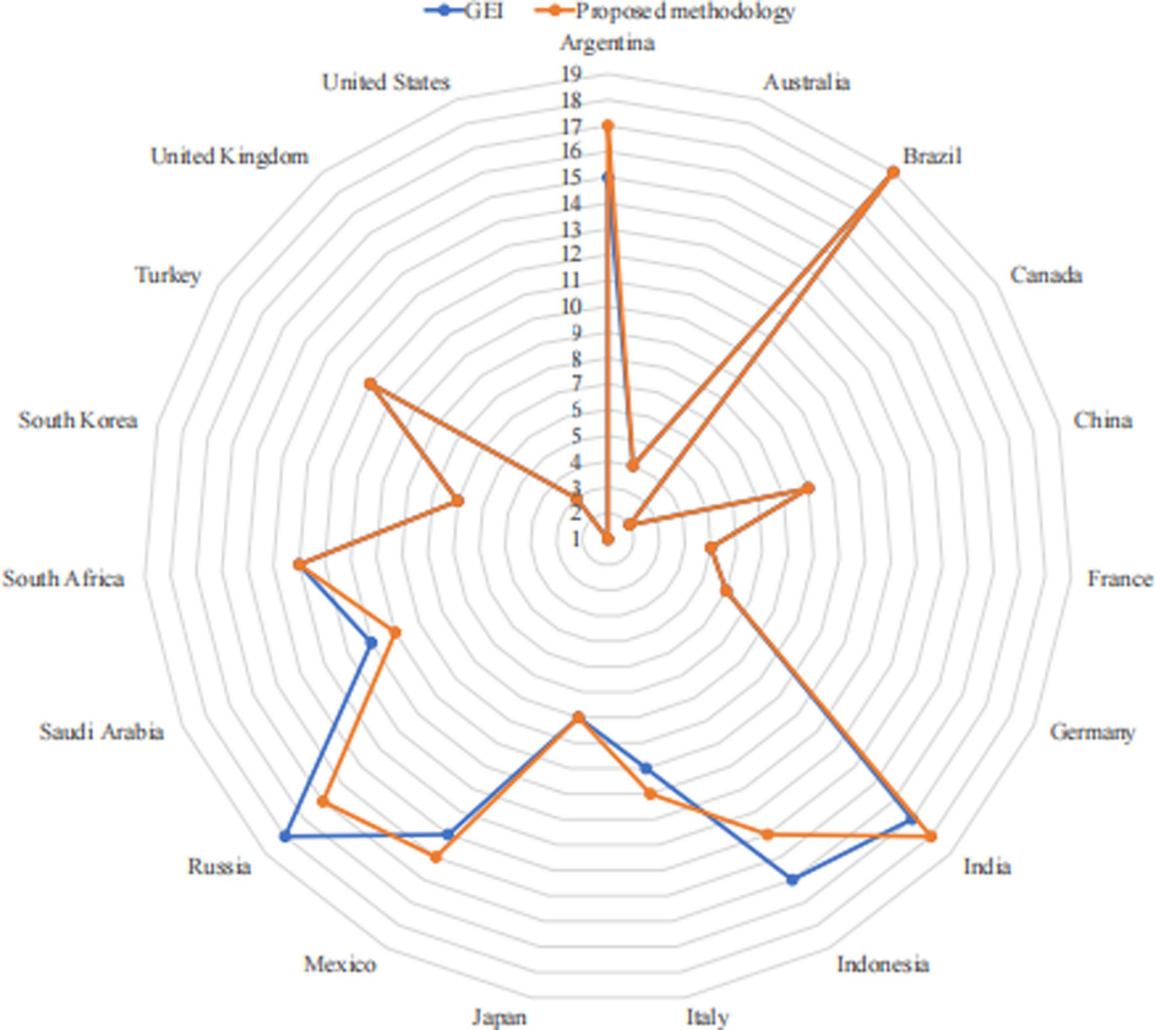
Ranking comparisons.

The ranking results obtained from different individual preferences: TBS, TSB, BTS, BST, STB, SBT and the proposed methodology are summarized in Table 7 and Fig 1. The ranking in accordance with the GEI is reported for the purpose of comparing as well. It is observed that 6 out of 19 countries are ranked differently. The obtained results depend on the BO and OW pairwise comparison vectors, which in a sense reveal the decision subjectivity. In terms of practical application, our methodology effectively takes the advantage of not only objective data, but also subjective decision preference. This is undoubtedly capable of increasing the decision flexibility and applicability.

**Table 7:** Ranking comparisons.

Country	TBS	TSB	BTS	BST	STB	SBT	GEI	Proposed methodology
Argentina	15	16	17	17	17	17	15	17
Australia	4	4	4	4	4	4	4	4
Brazil	19	19	19	19	18	18	19	19
Canada	2	2	2	2	2	2	2	2
China	9	9	8	9	10	10	9	9
France	5	6	5	5	6	6	5	5
Germany	6	5	6	6	7	7	6	6
India	18	18	14	15	19	19	17	18
Indonesia	14	14	16	16	13	13	16	14
Italy	10	10	10	10	11	11	10	11
Japan	7	7	7	8	9	9	8	8
Mexico	17	15	15	14	14	15	14	15
Russia	16	17	18	18	16	16	18	16
Saudi Arabia	11	11	12	12	8	8	11	10
South Africa	13	13	13	13	15	14	13	13
South Korea	8	8	9	7	5	5	7	7
Turkey	12	12	11	11	12	12	12	12
United Kingdom	3	3	3	3	3	3	3	3
United States	1	1	1	1	1	1	1	1

### 3.3. Sensitivity analysis

The results obtained are dependent on the pairwise comparison vectors, which reflect the decision subjectivity. In this regard, it is of great significance to investigate how the results would change if these two vectors change. Therefore, we perform a sensitivity analysis to show how much the proposed methodology is sensible to these subjective decisions. TBS and SBT are changed as the best and worst scenarios, respectively. The pairwise comparison vectors are shown in Table 8. The optimal weights are derived as $\lambda_{TBS}^{*}=0.3438$, $\lambda_{TSB}^{*}=0.1875$, $\lambda_{BTS}^{*}=0.1875$, $\lambda_{BST}^{*}=0.1250$, $\lambda_{STB}^{*}=0.0938$, $\lambda_{STB}^{*}=0.0625$. The ranks of 19 countries in this case are then obtained and compared (See Table 9). It is observed that when the best and worst scenarios are swapped, the ranks of these countries are changed mildly, in which only the ranks of 6 countries are changed with 1 ranking position.

**Table 8:** Best-to-Others (BO) and Others-to-Worst (OW) pairwise comparison vectors for sensitivity analysis.

BO: Best SBT	TBS	TSB	BTS	BST	STB	SBT
OW						Worst: TBS
TBS						5
TSB						3
BTS						3
BST						2
STB						2
SBT						1

**Table 9:** Sensitivity analysis.

Country	GEI	Before	After
Argentina	15	17	16
Australia	4	4	4
Brazil	19	19	19
Canada	2	2	2
China	9	9	9
France	5	5	5
Germany	6	6	6
India	17	18	18
Indonesia	16	14	14
Italy	10	11	10
Japan	8	8	7
Mexico	14	15	15
Russia	18	16	17
Saudi Arabia	11	10	11
South Africa	13	13	13
South Korea	7	7	8
Turkey	12	12	12
United Kingdom	3	3	3
United States	1	1	1

### 3.4. Policy implications

Our empirical study gives rise to important policy implications for the policy makers who are interested in designing and applying sophisticated entrepreneurship performance measurement method. On the one hand, the comprehensive consideration of all possible individual preferences among entrepreneurial attitudes, entrepreneurial abilities and entrepreneurial aspirations would yield different ranking with respect to entrepreneurship performance. This is essentially in line with the existing systemic rationale to measure the national entrepreneurship performance. On the other hand, it is crucial to incorporate the policy makers’ subjective judgement about the importance degree of each individual preference in evaluating the entrepreneurship performance. The sensitivity analysis indicates that different rankings would be resulted from different subjective judgements.

## 4. Concluding remarks

This paper proposes a new methodology based upon individual preference and BWM with entropy measures to modify the published Global Entrepreneurship Index, which alternatively considers all individual preferences among the sub-indices, namely, entrepreneurial attitudes, entrepreneurial abilities and entrepreneurial aspirations. Each individual preference is denoted by a mild weight restriction. Under each individual preference, we develop a sophisticated transformation to derive the performance of countries/regions, which could then be revised by means of calculating the entropy measures of them. The BWM is employed to generate a set of common weights associated with each individual preference to aggregate the results of each country/region. A case study is presented to show the effectiveness of our methodology.

The prominent advantage of our methodology is an integration of subjective judgements and objective measures, which could be further explored in future by developing other subjective and objective opinions to better characterize the decision problem. In addition, the entropy values are utilized to reduce the decision redundancy in this study, future research should consider other schemes to achieve this goal.

## Supporting information

S1 Data (XLSX)

## References

[1] Van Praag C M, Versloot PH. What is the value of entrepreneurship? a review of recent research. Small Business Economics. 2007, 29: 351–382. 10.1007/s11187-007-9074-x

[2] Van Stel A, Carree M, Thurik R. The effect of entrepreneurial activity on national economic growth. Small Business Economics. 2005, 24: 311–321. 10.1007/s11187-005-1996-6

[3] Valliere D, Peterson R. Entrepreneurship and economic growth: Evidence from emerging and developed countries. Entrepreneurship and Regional Development. 2009, 21: 459–480. 10.1080/08985620802332723

[4] Hessels J, van Stel A. Entrepreneurship, export orientation, and economic growth. Small Business Economics. 2011, 37: 255–268. 10.1007/s11187-009-9233-3

[5] Du K, Allan O’Connor. Entrepreneurship and advancing national level economic efficiency. Small Business Economics. 2018, 50(1): 91–111. 10.1007/s11187-017-9904-4

[6] Sutter C, Bruton GD, Chen J. Entrepreneurship as a solution to extreme poverty: A review and future research directions. Journal of Business Venturing. 2019, 34: 197–214. 10.1016/j.jbusvent.2018.06.003

[7] Zoltán J Ács Erkko Autio, Szerb László. National systems of entrepreneurship: Measurement issues and policy implications. Research Policy, 2014, 43(3): 476–494. 10.1016/j.respol.2013.08.016

[8] Bonyadi E, Sarreshtehdari L. The Global Entrepreneurship Index (GEI): a critical review. Journal of Global Entrepreneurship Research. 2021: 1–20. 10.1007/s40497-021-00302-0

[9] Ács Zoltán J., Szerb László, Raquel Ortega-Argilés, Coduras A, Aidis R. The regional application of the global entrepreneurship and development index (GEDI): the case of Spain. Regional Studies. 2015, 49(12): 1977–1994. 10.2139/ssrn.1970009

[10] Junior EI, Dionisio EA, Fischer BB, Li Y, Meissner D. The global entrepreneurship index as a benchmarking tool? criticisms from an efficiency perspective. Journal of Intellectual Capital. 2021, 22: 190–212. 10.1108/JIC-09-2019-0218

[11] Baker T, Gedajlovic E, Lubatkin M. A framework for comparing entrepreneurship processes across nations. Journal of International Business Studies. 2005, 36: 492–504. 10.1057/palgrave.jibs.8400153

[12] Marcotte C. Measuring entrepreneurship at the country level: A review and research agenda. Entrepreneurship and Regional Development. 2013, 25(3–4): 174–194. 10.1080/08985626.2012.710264

[13] Terjesen S, Hessels J, Li D. Comparative international entrepreneurship: A review and research agenda. Journal of Management. 2016, 42: 299–344. 10.1177/0149206313486259

[14] Wang TJ, Fu YL. Constructing composite indicators with individual judgements and best-worst method: An illustration of value measure. Social Indicators Research. 2020, 149: 1–14. 10.1007/s11205-019-02236-3

[15] Fu YL, Lai KK, Yu L. Multi-nation comparisons of energy architecture performance: A group decision- making method with preference structure and acceptability analysis. Energy Economics. 2021, 96, 105139. 10.1016/j.eneco.2021.105139

[16] Szerb László, Ács Zoltán J. The global entrepreneurship and development index methodology. Available at SSRN 1857985. 2011. doi: 10.1039/c0dt01842b 21451823

[17] Zoltán J Ács Rappai Gábor, Szerb László. Index-building in a system of interdependent variables: The penalty for bottleneck. GMU School of Public Policy Research Paper. 2011. 10.2139/ssrn.1945346

[18] Melkonyan T, Safra Z. Intrinsic variability in group and individual decision making. Management Science. 2016, 62(9): 2651–2667. 10.1287/mnsc.2015.2255

[19] Lai KK, Ng WL. A stochastic approach to hotel revenue optimization. Computers and Operations Research. 2005, 32(5): 1059–1072. doi: 10.1016/j.cor.2003.09.012

[20] Shannon CE. A mathematical theory of communication. ACM Sigmobile Mobile Computing and Communications Review. 2001, 5: 3–55.

[21] Rezaei J. Best-worst multi-criteria decision-making method. Omega. 2015, 53: 49–57. 10.1016/j.omega.2014.11.009

[22] Rezaei J. Best-worst multi-criteria decision-making method: Some properties and a linear model. Omega. 2016, 64: 126–130. 10.1016/j.omega.2015.12.001

[23] Saaty TL. Axiomatic foundation of the analytic hierarchy process. Management Science. 1986, 32: 841–855. 10.1287/mnsc.32.7.841

